# Neuroimaging at Term Equivalent Age: Is There Value for the Preterm Infant? A Narrative Summary

**DOI:** 10.3390/children8030227

**Published:** 2021-03-16

**Authors:** Rudaina Banihani, Judy Seesahai, Elizabeth Asztalos, Paige Terrien Church

**Affiliations:** Newborn & Developmental Paediatrics, Sunnybrook Health Science Centre, 2075 Bayview Ave, The University of Toronto, Toronto, ON M4N 3M5, Canada; judy.seesahai@sunnybrook.ca (J.S.); elizabeth.asztalos@sunnybrook.ca (E.A.); paige.church@sunnybrook.ca (P.T.C.)

**Keywords:** neonates, magnetic resonance imaging, neuroimaging, cranial ultrasound, neurodevelopmental, premature infants, parental perception

## Abstract

Advances in neuroimaging of the preterm infant have enhanced the ability to detect brain injury. This added information has been a blessing and a curse. Neuroimaging, particularly with magnetic resonance imaging, has provided greater insight into the patterns of injury and specific vulnerabilities. It has also provided a better understanding of the microscopic and functional impacts of subtle and significant injuries. While the ability to detect injury is important and irresistible, the evidence for how these injuries link to specific long-term outcomes is less clear. In addition, the impact on parents can be profound. This narrative summary will review the history and current state of brain imaging, focusing on magnetic resonance imaging in the preterm population and the current state of the evidence for how these patterns relate to long-term outcomes.

## 1. Introduction

Advances in perinatal and neonatal care have improved survival rates for very preterm infants (born ≤ 30 weeks gestation age GA) [1,2,3]. The developing preterm brain is uniquely vulnerable to hypoxic, hemorrhagic, and/or inflammatory injury. The two most frequently reported findings include intracranial hemorrhage and white matter abnormalities (WMA) [4,5]. Both are associated with substantial neurodevelopmental challenges, including cerebral palsy (CP) and mild motor dysfunction, neurosensory impairment, cognitive, language, as well as behavioural disorders such as attention-deficit–hyperactivity disorder (ADHD) and autism spectrum disorder (ASD) [6,7,8]. These outcomes have associated impacts on individuals, families, and society [9]. Early identification and the provision of targeted interventions are essential to the infants’ and their families’ quality of life [10,11,12,13].

As neuroimaging has evolved, the identification of brain injuries in the preterm population has become more sophisticated, demonstrating a complex and diverse spectrum of brain injury [14]. Ironically, this diverse pattern of presentations on neuroimaging has limited the prediction of outcome [15]. The univariate nature of imaging is one of the most significant limiting factors for its prognostic efficacy. Images alone cannot account for the complex nature of perinatal and neonatal brain injuries with a potpourri of extracranial factors correlated to the developmental outcome, including nutrition, social and economic factors, and access to early identification and targeted interventions [16]. Additionally, the dynamic nature of brain development and potential injury in this unique population underlies the significance of the timing of imaging. These collective factors contribute to the widely variable sensitivity and specificity of neuroimaging in predicting later development.

This narrative summary will review the history and current state of brain imaging, focusing on cranial ultrasound and magnetic resonance imaging (MRI) in the preterm population to predict later neurodevelopmental outcomes. The updated guidelines from the American Academy of Neurology (AAN) [17], the American Academy of Pediatrics (AAP), and the Canadian Pediatric Society (CPS) [5,18] regarding MRI screening in this population will also be summarized.

## 2. History of Neuroimaging and Patterns of Brain Injury in the Preterm Population

Neuroimaging emerged as a diagnostic tool in the mid-1970s with cranial ultrasonography (US) to assess intracranial hemorrhage in neonates [19]. In 1978, Dr. Volpe indicated that one of the primary goals of neuroimaging in preterm infants was to “identify those with a hopeless prognosis” [20]. Neuroimaging offered additional depth and detail previously unavailable.

Over the last 40 years, cranial US, with its ease of use, has become the primary neuroimaging modality to evaluate intracranial pathology in preterm infants and predict long-term neurodevelopmental outcomes. It is cost-efficient, readily available, and easily performed at the bedside. In addition, it offers an imaging modality free of exposure to ionizing radiation as opposed to computerized tomography (CT) scans [21]. A routine imaging schedule for preterm infants less than 32 weeks gestational age (GA) with the cranial US is now followed in neonatal intensive care units (NICU) [5,18]. In order to standardize the descriptions of injury documented on the cranial US, grading systems were developed by Papile et al. [22] Table 1 and Volpe [23], with that from Papile being the most widely used to date.

One key change from Volpe, however, has been the revision of the finding once referred to as a Grade IV IVH by Papile [22] to that which is now referred to as a periventricular hemorrhage (PVH) [23]. A PVH infarction (PVHI) is attributed to impaired venous drainage of the white matter’s medullary veins following a GMH-IVH [24,25,26]. It is viewed as a separate finding from IVH and can be found with any injury grade. In addition to hemorrhagic injuries, cranial US also provided visualization of ischemic injury of the cortical white matter, referred to as periventricular leukomalacia (PVL) [26,27,28]. Expansion of the intracranial windows has also allowed greater visualization of the cerebellum, now recognized as having the potential for hemorrhagic or ischemic injury and its own consequences for neurodevelopmental outcomes [29].

Imaging the entire brain was once performed using computer tomography (CT), but this has shifted to cranial US and MRI, which offer either more specific detail and avoid ionizing radiation [30]. One of the earliest studies using CT scans in preterm was in 1983 to predict long-term outcomes in the preterm population. The authors described the association of major developmental and neuromotor handicaps with the findings of more severe (grade III and IV) IVH on CT scans performed between 3 and 10 days of age [31]. In 1994, infants with HIE were receiving at least one CT scan during their initial hospitalization as it was deemed more efficient, despite concerns regarding radiation exposure, the ease of cranial US, and the superiority of MR imaging in this population [32]. CT can identify severe deep gray matter lesions with similar sensitivity to MRI, and injuries to the thalami and basal ganglia on CT have been associated with death or significant neurologic sequelae at 18 months of life [33]. However, CT does not identify white matter cortical injury or cerebellar injury as effectively as MRI [34,35]. CT has been used to assess for calcifications, hemorrhage, brain injury, and edema secondary to hypoxia-ischemia, venous sinus thrombosis, masses, and structural abnormalities, but this modality is now primarily supplanted by MRI due to the ionizing radiation required for imaging. Except for emergencies, CT scans are now generally avoided for newborn imaging [5]. Therefore, CT is no longer considered a part of routine imaging techniques of the preterm brain. The main benefit of CT remains its rapid acquisition time, obviating the need for sedation of the infant and better visualization of superficial structures over the cranial US.

In 1985, MRI emerged as an additional safe imaging option for neonatal brains [36], initially describing findings in neonatal encephalopathy (NE) [37]. Benefits associated with MRI included more detailed images and no ionizing radiation [38,39,40,41]. Since those early studies, MRI technology has demonstrated better sensitivity and specificity for detecting brain injury in neonates, particularly in the thalami and basal ganglia [42]. The use of MRI extended into the preterm cohort as well, demonstrating a broader sense of the extent of white matter injury or white matter abnormality (WMA) [43]. Types of WMA include ventriculomegaly, decreased white matter volume, increasing intensity of white matter signal, and evidence of decreased myelination [44,45,46,47]. The remainder of the MRI abnormalities described in preterm infants are more focal in nature, most commonly being punctate lesions [48,49,50], representing clusters of activated microglia. Although classic cystic PVL is the WMA that has been the most thoroughly investigated in preterm infants, it represents only about 4% of the abnormalities seen on term equivalent age (TEA) MRI [48,49].

Another significant advantage of MRI is the detailed images of the posterior fossa and detection of cerebellar hemorrhage (CBH), ranging from small punctate lesions, focal unilateral bleeds, and massive bleeds involving both hemispheres and including the vermis [51,52]. While few IVH are missed by routine cranial US, CBH is identified more readily on MRI versus cranial US [44,47,53]. A recent study showed CBH on MRI in 10% of the cohort, whereas only 2% had hemorrhage detected on the cranial US [54,55]. A grading system for CBH has been developed based on the lesion(s) location and extent of the bleeding as crucial variables concerning the neurodevelopmental outcomes [47,52,56,57].

Practical considerations include the cost of MRI estimated at GBP 315 (CAD 550) per patient [38,58]. There are also technical challenges, with MRI not being available in most NICUs at the bedside, requiring transportation to the imaging suite. They are also more time consuming to obtain and more sensitive to motion artifacts, thereby requiring sedation. Sedation and neonatal transport are often imperative, requiring personnel with these competencies [59,60,61,62,63].

In addition, there are an array of contraindications to MRI, including implanted or attached electronic and ferromagnetic devices (e.g., pacemakers, ferrous aneurysm clips). All personnel, monitoring and support equipment must be safe for the magnet. Yet, most resuscitation equipment is not magnet-safe and cannot be brought into the MRI scanning room, limiting the modality to those that are clinically stable or for whom there is no other acceptable option. [39,64]. Lastly, accurate neonatal MRI readings require expertise by knowledgeable pediatric neuroradiologists. In particular, the ability to detect mild and moderate degrees of injury on MRI may need sophisticated scanning sequences as well as additional proficiency in the analysis of these results [65,66,67].

## 3. Correlation of Neuroimaging Findings with Neurodevelopmental Outcomes

These advances in the development and utilization of cranial US and MRI have enhanced brain injury detection in preterm infants and improved the understanding of the links between brain injury and neurodevelopmental outcomes. In particular, neuroimaging is able to identify preterm infants with significant brain injury who are at-risk for neurodevelopmental challenges [15,44,68]. This section will review the specific imaging technique and evidence around the associated long-term outcome.

### 3.1. Cranial US

Cranial US remains the NICU’s primary imaging tool worldwide [5,18,56]. This is related to its ease of use and cost-efficiency. It has also been the most commonly used neuroimaging modality for predicting long-term outcomes [69].

The correlation between IVH diagnosed by the cranial US in the first one to two weeks of life and later developmental outcomes were the focus of early studies. One of the earliest outcome studies in 1989 reported that grade II or higher cerebroventricular hemorrhage (CVH) was 79% sensitive for the development of CP and 70% sensitive for intellectual challenges by two years of life [70]. Today, however, CP’s risk due to isolated IVH has been much lower, mostly due to care and management changes in the NICU [71,72]. Subsequent recent studies, including extremely low gestational age neonates (ELGANs), have estimated the risk rate of CP in isolated IVH to be between 9 and 17%, compared to 4–6% in infants with normal cranial US results [73,74]. The finding of PVHI (described originally as grade IV IVH) has been associated with more significant motor and cognitive impairments [75,76,77,78,79,80]. PVL was reported to be the strongest sonographic predictor of abnormal motor outcomes and CP in preterm infants [81,82,83,84,85].

Late cranial US findings (35 to 42 weeks postmenstrual age) associated with cognitive delay and/or psychomotor delay include moderate/severe ventriculomegaly, echolucencies and echodensities, severe IVH (grade III or higher), and periventricular hemorrhage [15,69,79,86].

The predictive value of cranial US remains low despite technological advances and high-frequency probes. The ELGAN study found 43% of infants who develop CP had no significant IVH, WMA, or ventriculomegaly on the cranial US, and 6% had normal findings on the cranial US [74]. Table 2 presents a review of some of the evidence around the cranial US and long-term outcome.

**Table 2:** The predictive value of the cranial ultrasound in preterm infants.

Study/Year	Year of Recruitment	Population Characteristics GA in Weeks (toTal Number of Infants)	Ages of Assessment	Lesions with the Highest Correlation	Outcome Measure	Age of Outcome Measure (Corrected Age when Age in Months)	Predictive Result			
							Sens (%)	Spec (%)	PPV (%)	NPV (%)
O’Shea et al., 2008 (ELGAN) [69]	2002–2004	<28 (1506)	Variable—day 1 and 4 or day 5 and 14 or day 15 to 40th postconceptional weeks or a combination of all above	V.E./Echolucent lesion	MDI +/−VABS ABC < 70	24 months	12–17	93–95	45	75–76
					PDI of <70 +/−VABS ABC < 70		14–17	94–96	55–61	71–72
Kuban et al., 2009 (ELGAN) [74]	2002–2004	(1105)	Variable—day 1 and 4 or day 5 and 14 or day 15 to 40th postconceptional weeks or a combination of all above	V.E./Echolucent lesion	CP	24 months	32–38	94–96	44–52	>92
Leijser et al., 2008 [87]	May 2001–Apr 2004	<32 (40)	Average of 7 US between day of birth until discharge or transfer, and TEA	Major Lesions ^a^	BSID II, MDI, PDI) of <70	24 months	75	86	43	96
Woodward et al., 2006 [68]	November 1998–May 2002	≤30 (1962)	Minimal by 48 h of life, at 5 to 7 days of age, and again at 4 to 6 weeks of age	Major Lesions ^a^	CP	24 months	18	85	-	-
De Vries et al., 2004 [88]	January 1990–January 1999	<32 (1460)	Weekly until discharge and 40 weeks PMA	Major Lesions ^a^	CP	24 months	76	95	48	99
		33–36 (469)	Weekly until discharge and 40 weeks PMA	Major Lesions ^a^	CP	24 months	86	99	83	99
Valkama et al., 2000 [89]	November 1993–October 1995	<34 (51)	Term	Major Lesions ^a^	CP	18 months	67	85	-	-
Pinto-Martin et al. 1995 [90]	September 1984–June 1987	(1105)	4 and 24 h and 7 days of life; with 47% also scanned in week 5 and/or Predischarge	PEL/VE	Disabling CP	24 moths	54	96	-	-
Nwaesei et al.1988 [91]	July 1984–June 1985	≤32 (110)	US at 1 week	Major Lesions ^a^	CP or BSID III < 85	12 months	16	99	75	85
			US at 2 weeks				16	99	75	85
			US at 3 weeks				37	99	87	87
			US at 6 weeks				53	99	91	91
			US at 40 weeks PMA				58	100	100	92
Graham et al. 1987 [92]	January 1984–April 1985	Selected on weight ≤ 1500 g, not GA (200)	At least twice weekly for the first month and then every week until discharge.	PVH	CP	18 months	67	53	11	95
				Cystic PVL			67	96	62	97
				Prolonged Flare			17	85	9	92

Note. ^a^ Major lesions: Grade III-IV IVH, cystic PVL: subcortical leukomalacia, basal ganglia lesions, or focal infarction. ABC: adaptive behavior composite, BSID: Bayley Scales of Infant and Toddler Development, CP: cerebral palsy, DWMA: diffuse white matter abnormality, ELGAN: extremely low gestational age newborns, MABC: Movement Assessment Battery for Children, MDI: Mental Developmental Index. MRI: magnetic resonance imaging, NPV: negative predictive value, PDI: Psychomotor Developmental Index, PEL/VE: parenchymal echodensities/lucencies or ventricular enlargement, PL/VE: parenchymal lesions/ventricular enlargement, PPV: positive predictive value, PVH: periventricular hemorrhage, PVL: periventricular leukomalacia, Sens: sensitivity, Spec: specificity, TEA: term equivalent age, US: ultrasound, weeks: weeks, VABS: Vineland Adaptive Behavior Scales, WMA: white matter abnormalities.

### 3.2. Magnetic Resonance Imaging

In contrast, MRI has demonstrated more sensitive and specific imaging information about central nervous system (CNS) abnormalities. As a result, MRI is increasingly being used in many NICU settings to identify cerebral WMA in preterm infants’ brains at TEA [93,94,95].

One of the first extensive studies to examine the link between findings on neuroimaging and neurodevelopmental outcomes was by Woodward et al. [68], which demonstrated improved MRI sensitivity over the cranial US in predicting a range of neurodevelopmental challenges. The sensitivity associated with moderate-to-severe WMA on MRI for predicting cognitive delay at two years was 41%, and 65% for severe motor delay or CP, respectively. Subsequent studies (see Table 3) have demonstrated a correlation between neurodevelopmental outcome and either grossly abnormal or normal TEA MRI scans [4,15,44,46,68,96,97]. For those extremely preterm infants (GA < 28 weeks) with a normal TEA cranial US, studies have demonstrated that there is a low likelihood of finding moderate or severe white matter or gray matter abnormalities on TEA MRI [98,99,100]. As a complement to the cranial US, TEA MRI has emerged to improve prognostic information and inform current clinical and future supportive care [101]. Table 3 presents a review of individual studies on MRI and their associated long-term outcome.

**Table 3:** The predictive value of the MRI at TEA in preterm infants.

Study/Year	Year of Recruitment	Population Characteristics GA in Weeks (Total Number of Infants)	Lesion with Highest Correlation	Outcome Measure	Age of Outcomes Measures (Corrected Age when Age in Months)	Predictive Result			
						Sens (%)	Spec (%)	PPV (%)	NPV (%)
Parikh et al., 2020 [102]	November 2014 and march 2016	≤31 (98)	Moderate-to-severe DWMA	BSID III Cognitive < 70	24 months	100	95.7	-	-
				BSID III language < 70		37.5	93.9		
Slaughter et al., 2016 [103]	August 2005 and November 2007	Based on the weight of ELBW, not GA (122)	Diffuse cystic changes	Death or CP	18–24 months	33	94	-	-
			Gyral maturational delay	Death, CP, BSID III < 80, or sensory challenges (vision or hearing loss)		33	97		
Spittle et al., 2011 [104]	2001 and 2003	<30 weeks or birthweight < 1250 g (227)	Moderate-to-severe WMA (30)	CP or MABC < 5th percentile	5 years	-	-	34	91.4
			Any Severity WMA					92.5	40.7
Woodward et al., 2006 [68]	November 1998–May 2002	≤30 (1962)	Moderate-to-severe WMA in (35) 21%	CP or severe cognitive or motor delay	24 months	41–65	84–85	-	-
			Any Severity WMA			88–94	30–31		
Valkama et al., 2000 [89]	November 1993–October 1995	<4 (51)	Parenchymal lesions: PVH, PVL, or infarct WMA	CP	18 month	100	79	-	-

ABC: adaptive behavior composite, BSID: Bayley Scales of Infant and Toddler Development, CP: cerebral palsy, DWMA: diffuse white matter abnormality, ELGAN: extremely low gestational age newborns, MABC: Movement Assessment Battery for Children, MDI: Mental Developmental Index. MRI: magnetic resonance imaging, NPV: negative predictive value, PDI: Psychomotor Developmental Index, PPV: positive predictive value, PVH: periventricular hemorrhage, PVL: periventricular leukomalacia, Sens: sensitivity, Spec: specificity, TEA: term equivalent age, US: ultrasound, weeks: weeks, VABS: Vineland Adaptive Behavior Scales, WMA: white matter abnormalities.

A meta-analysis by George et al., 2018 examined data from 31 articles to evaluate the diagnostic accuracy of early MRI performed before 36 weeks postmenstrual age in relation to later motor outcomes and CP. The results demonstrate that early structural MRI had a sensitivity of 100% and specificity of 93% for the identification of children with CP [105]. These results reinforce those from Van’t Hooft et al., 2015, which demonstrated a similar CP prediction result [106]. However, prognostic accuracy for visual and/or hearing problems, neurocognitive and/or behavioural function was poor [106].

Imaging the posterior fossa and detecting CBH is one of the significant advantages of MRI over cranial US [29,55]. CBH has been associated with a substantial risk of neurologic abnormalities in preterm infants [107,108,109,110]. A follow-up study of infants with CBH at seven years of age found that these children have more challenges with attention [111,112,113]. Disrupted cerebellar development has been linked to a future diagnosis of autism spectrum disorder (ASD) [114], and other psychiatric disorders (e.g., schizophrenia) [115,116]. A high incidence of nonmotor (cognitive, language, and behavior development) delay in infants with neonatal CBH was confirmed in a recent systematic review by Hortensius, Dijkshoorn et al., 2018 [109].

### 3.3. Clinical Implications of Imaging at TEA

There is evidence that abnormal findings on neuroimaging can aid in the prediction of the long-term neurodevelopmental outcome, but the challenge lies in that the predictive value is unclear, higher for more obvious lesions and lower for less clear findings (see Table 2 and Table 3). The correlation between TEA cranial US and TEA MRI has a consistency of up to 88% for the predictive value in very preterm infants with brain injury evaluated for neurodevelopmental outcomes at two years of age [117,118]. In addition, up to 25% of preterm infants with an unremarkable exam on the cranial US may still present later in childhood with cognitive or psychomotor delays [69]. A similar dilemma applies to MRI studies, as not all children with WMA at TEA MRI had significant challenges, and profound neurologic impairment occurred in other children without WMA. Table 4 presents a review of some studies with a normal or unremarkable exam on neuroimaging (cranial US and MRI) [99].

**Table 4:** Normal neonatal imaging (Cranial US and TEA MRI) exam and neurodevelopmental outcomes in preterm infants.

**Cranial US.**											
**Study/Year**	**Year of Recruitment**	**Population with no US Abnormalities**	**Ages of Assessment**	**Corrected Age of Outcomes Measures**	**Outcome**						
					**Cognition**		**CP (%)**	**HI. (%)**	**VI (%)**	**NDI (%)**	**Other (%)**
					**BSID MDI < 70 (%)**	**BSID PDI < 70 (%)**					
Hou et al., 2020 [119]	2005 to 2010	BW < 1250 g (*n*) 192	Serially from birth until Term	2 years	22.4 BSID III < 80	-	2.1	-	-	-	-
Munck et al., 2010 [120]	2001 to 2006	VLBW infants BW < 1500 g (*n*) 91	Serially at 3–5 days, 7–10 days, at 1 month and then monthly discharge and then at term	2 years	2 BSID II	-	0	0	-	2	ID 2
Kuban et al., 2009 (ELGAN) [74]	2002 to 2004	<28 weeks infants (*n*) 739	Variable—day 1 and 4 or day 5 and 14 or day 15 to 40th postconceptional weeks or a combination of all above	2 years	-	-	6	-	-	-	-
Laptook et al., 2005 [121]	1995 to 1999	GA 26 +/− 2 weeks BW < 1000 g infants (*n*) 1473	mean age of 6 and 47 days	18 to 22 months	25 BSIDII	-	9	-	-	29	ID 25
Adams-Chapman et al., 2008 [122]	1993 to 2002	BW 401–1000 g infants (*n*) 5163	n.s.	18 to 22 months	27 BSID IIR	17 BSID IIR	10	1	9	35	ID 27
Ancel et al., 2006 (EPIPAGE) [73]	1997	GA 22 and 32 weeks infants (*n*) 1238	1 to 3 times in the first 2 weeks of life and then every 2 weeks	2 years	-	-	4.4	-	-	-	-
Patra et al., 2006 [123]	1992 to 2000	GA 26.5 weeks ± 1.9 infants (*n*) 258	at least 2 in the first 10 days of life, then 30 days and at least 1 before discharge	20 months	25 BSIDII	28 BSID-II	3	2	-	28	ID 25
Sherlook et al., 2005 [124]	1991 to 1992	GA < 28 weeks BW < 1000 g infants (*n*) 180	At least 1 by 1st week of life, at 28 days, and prior to discharge	8 years chronological age	-	-	6.7	-	-	-	Low reading 24.4% Low spelling 19.2% Low arithmetic 27.6%
Whitaker et al. 1996 [125]	1984 to 1987	GA 32.1 ± 3.0 BW 501 to 2000 g infants (*n*) 468	4 and 24 h and 7 days of life; with 47% also scanned in week 5 and/or Predischarge	6 years	-	-	-	-	-	-	ID1.3
**TEA MRI**											
**Study/Year**	**Year of recruitment**	**Population with no US abnormalities**		**Age of outcomes measures**	**Outcome**						
					**Cognition**		**CP (%)**	**HI. (%)**	**VI (%)**	**NDI (%)**	**Other (%)**
					**BSID MDI < 70 (%)**	**BSID PDI < 70 (%)**					
Anderson et al., 2017 [97]	2001 to 2003	60 infants GA < 30 weeks BW < 1250 g		7 years corrected age	-	-	-	-	-	-	Intelligence quotient 100.2 (14.7) Mean (SD.) Motor 9.5 (3.7) Mean (SD.)
Munck et al., 2010 [120]	2001 and 2006	182 infants BW < 1500 g		2 years corrected age	-	-	2	-	-	2	ID 0
Woodward et al., 2006 [68]	1998 to 2002	GA < 30 weeks	No WMA (*n*) 47	2 years corrected age	4	-	2	-	-	15	ID 7
			No GrMA (*n*) 85		-	-	5	-	-	21	1D 10

BSID: Bayley Scale of Infant Development, BW: birth weight, EPIPAGE: Etude épidémiologique sur les petits âges gestationnels, HI: hearing impairment, ID: intellectual disability GA: gestational age, GrMA: gray matter abnormality, NDI: neurodevelopmental impairment, *n*: number of population, n.s.: not stated, MRI: magnetic resonance imaging, US: ultrasound, VLBW: very low birthweight, VI: vision impairment, Wks: weeks, WMA: white matter abnormality.

Term age equivalent MRI alone poorly predicted cognitive function for the individual patients at school age or later in life [97,126]. Adding TEA MRI to early and late cranial US also did not appear to improve the predictive ability of severe intellectual disability (ID) or significant neurodevelopmental challenges at six or seven years of age [15,97].

As a result of the challenges associating findings or lack thereof with long-term outcomes, neuroimaging (cranial US and/or MRI) does not yet offer the solitary accurate predictor of long-term neurodevelopmental outcomes for individuals. Limitations may relate to the use of early CP as a binary variable in this population; this is a primitive metric, as many infants with IVH who demonstrate signs of CP at one to two years of life have a minimal functional impairment and an overall intelligence similar to that of controls by the time they reach school age [127]. In addition, it endorses a misperception that PVL and PVH carry universally ‘poor’ outcomes, which may potentially lead to alterations of care or withdrawal of care [128]. Complex decisions such as withdrawal of support necessitate a deeper investigation into models of developmental outcomes, including an emphasis on factors such as functional impacts, family impact, and quality of life; this does not yet exist [58].

## 4. Effect of Diagnostic MRI on Parents and Further Follow-Up

Preterm birth has long-lasting effects on individuals and families, and increased maternal anxiety adversely influences child development [129,130]. Based on data to date, neuroimaging’s predictive capabilities for high-risk infants are inadequate to identify those infants that should be excluded from structured follow-up or those that should be selected for additional therapies [131,132,133]. Additionally, the imaging technique with the greatest predictive capacity, MRI, is performed after the acute phase of illness, making it unavailable for counselling parents on limitations of care or support withdrawal [134,135]. The main remaining benefit is that of providing parents with a risk-adjusted estimate of the developmental outcome, something that has been suggested by families and physicians to be of questionable benefit and potentially emotionally harmful [134,136]. Pearce and Baardsnes (2012) articulated parental perspective in the impact of neuroimaging at TEA [136]:

“*We are not angry at the hospital, but knowing what we know now, we never would have consented to an MRI, because it served no purpose other than to traumatize a family that had already been through so much and affect our ability to enjoy bonding with our child*.”

Subsequently, parents have reported that there is a significant emotional impact associated with brain imaging during their infant’s NICU admission [58,136]. Edwards, Redshaw et al., 2018, studied the effect of MRI on preterm infants and their families and demonstrated that maternal anxiety was reduced after receiving information from neuroimaging (cranial US and/or MRI) and slightly more after an MRI compared with the cranial US. However, this reduction was not clinically significant and did not contribute to a better health-related quality of life for the child and family [58].

While the evidence is limited [137], most parents have expressed interest in more accurate long-term prognostic information, especially from a TEA MRI, in order to properly plan post-discharge early intervention support services. Despite that, it has been reported that parent’s concerns about long-term developmental outcomes and the need for information did not diminish over time or with the knowledge of assuring normal brain neuroimaging results and the baby’s stable condition [137,138,139]. The emotional impact of having a preterm baby had a negative effect on parents’ ability to retain information while in the NICU, and all had an ongoing need for reassurance beyond the hospital period [137].

In addition to the parent’s perspective regarding neuroimaging in the NICU, 10% of infants will demonstrate unexpected abnormalities on TEA MRI not acquired in the perinatal period (i.e., not already diagnosed prenatally or postnatally by clinical signs or the cranial US) [140,141]. Incidental findings in the adult population are identified in 1–4% of brain MRIs, with up to one-third of those neoplastic. Many of the remaining findings were considered benign without the need for follow-up [142]. Contrary to adult literature, extreme preterm infants tend to have a higher rate of incidental findings (9.7%) on TEA MRI requiring follow-up [140,141]. These incidental findings increase parental anxiety [143,144]. One-quarter of these findings were deemed benign (e.g., small benign venous anomalies, arachnoid cysts, corpus callosum dysgenesis, absent septum pellucidum, frontal scalp mass and a nasal septum cyst) and required no further follow-up. The remaining three-quarters required further diagnostic follow-up and/or therapeutic intervention, including cortical tubers, significant dysmorphia of the brain stem or cerebellum, and ectopic pituitary [140,141]. The balance between revealing silent brain abnormalities of clinical significance and discovering findings of uncertain clinical importance that result in potentially unnecessary diagnostic follow-up has led to the ongoing debate regarding the benefit and appropriateness of TEA MR imaging in this population [134,143].

## 5. Moving from Research to Clinical Practice

Despite the lack of clarity in the data as well as the cost, there have been calls to standardize the use of MRI for all former extreme preterm infants at 36–40 weeks postmenstrual age [145,146,147,148]. Magnetic resonance imaging has significant predictive value as an isolated imaging modality. However, several issues must be overcome before TEA MRI can become a standard in preterm infants. The current MRI data limit TEA MRI’s clinical usefulness to predict the long-term neurodevelopmental outcome for the individual survivor accurately [44]. In addition, there are no data to support that any improved prognostic capabilities of MRI will translate into improved outcomes. Due to these limitations, performing TEA MRI for preterm infants was recently listed as one of the top five tests or treatments in newborn medicine that “cannot be adequately justified based on efficacy, safety, or cost” [134,149].

Recommendations from the American Academy of Neurology (AAN), the American Academy of Pediatrics (AAP), and the Canadian Pediatric Society (CPS) do not endorse routine MRI for preterm infants, regardless of cranial US findings [5,17,18]. Currently, updated guidance from both the AAP and CPS state that TEA MRI for infants should be considered mainly as a follow-up of abnormal cranial US results (e.g., severe IVH, PVL, and hydrocephalus) and following a conversation with the family regarding its limitations for estimation of long-term prognosis. [5,18,105,134,136]. A “feed and wrap” technique is preferred should MRI be pursued [18,61,150]. Brain CT is no longer considered a part of routine imaging techniques for the preterm population [18].

While MRI has not yet emerged as a solitary tool, its role as contributing important data cannot be overlooked. It is for this reason that there is a consideration for TEA MRI to be integrated into a more extensive assessment, including physical examination findings, clinical risk factors (e.g., neonatal history of bronchopulmonary dysplasia and postnatal steroids) [151,152], standardized neurological and motor assessments (e.g., General Movement Assessment (GMA) [153,154,155], Hammersmith Infant Neurological Examination (HINE)) [155,156,157,158] for early identification of motor challenges, mainly CP [159,160,161,162,163]. In a retrospective case–control study of 441Italian high-risk infants, Morgan et al., 2019 found that the combined predictive power of the three-month HINE, GMA, and neuroimaging (MRI and/or cranial US) post-term gave sensitivity and specificity values of 97.86% and 99.22% (PPV 98.56%, NPV 98.84%) for detecting CP [164]. The clinical combination of these tools leads to earlier identification of CP diagnosis and facilitates earlier intervention. This will have significant implications for optimizing the child’s overall function [165,166], preventing secondary complications [167] and supporting the families of these children [168].

Two systematic reviews further supported a combined approach for the prediction of CP in conjunction with clinical history. Bosanquet et al., 2013 [169] looked at it in preschool-age children less than five years; one sampling mostly from preterm infants. Novak et al., 2017 [170] looked for clinical signs and CP symptoms that emerge and evolve before age two years, pooled from six systematic reviews and two evidence-based clinical guidelines. A more recent systematic review by Caesar et al., 2020 [171] evaluated the accuracy of clinical tools used at a corrected age of six months or younger to predict motor and cognitive delay at two years (not cerebral palsy) in infants born very preterm. Table 5 shows the result of the three recent systematic reviews of the predictive value of diagnostic tools that can identify infants at risk of long-term neurodevelopmental challenges [169,170,171].

This information is now being used by programs and guides screening tools, management regarding the intensity and duration of follow-up and the need for early identification and intervention. One case series suggested that this multivariate approach (risk factor assessment and MRI evidence of WMA) may help identify specific cognitive and behavioural problems in children born very preterm [172,173]. This is particularly challenging because available tools (neuroimaging and early clinical assessment) do not accurately predict long-term developmental outcomes such as cognitive, language, and behavior development in individual survivors [173], compared to predicting CP and/or motor challenges; see Table 2 and Table 3.

## 6. Conclusions

Prediction of neurodevelopmental outcomes is an essential aspect of neonatal care [13]. It allows for important counselling to offer essential information on the potential impact on a family, the child’s function, and future. It also allows for early identification and targeted intervention, which has been demonstrated to improve functional outcome [170]. Currently, however, there remains a critical gap in the research surrounding neonatal neuroimaging. While neuroimaging has not yet proven to be a tool that can be used in isolation, it has been proven to add value in combination with clinical history and examination. What remains to be articulated is whether the added value of TEA MRI details and sensitivity can be harnessed more effectively to outweigh the significant barriers to use, specifically cost, technical challenges, and expertise. In addition, or perhaps expanding on that statement, consideration is needed for a shift from a simple description of images and outcomes to that which explores how these tools can be leveraged to improve outcome, rather than predicting a ‘hopeless prognosis’.

## Figures and Tables

**Table 1:** Germinal matrix hemorrhage and intraventricular hemorrhage (GMH-IVH) grading system by Papile et al. [22].

Grade	Description in the Parasagittal View
I	Germinal matrix hemorrhage (GMH) only or germinal matrix hemorrhage plus intraventricular hemorrhage less than 10% of the ventricular area
II	GMH and intraventricular hemorrhage; 10 to 50% of the ventricular area
III	GMH and intraventricular hemorrhage involving more than 50% of the ventricular area; lateral ventricles are usually distended
IV	Hemorrhagic infarction in periventricular white matter ipsilateral to intraventricular hemorrhage (also called periventricular hemorrhagic infarction [PVHI])

Note. The description is in part based on the percentage (%) volume of the lateral ventricle fill by blood.

**Table 5:** The predictive value of tools used in the early (less than six months corrected age) identification of infants at risk of long-term neurodevelopmental challenges.

Study/Year	Country	Population Characteristics	Outcomes	Age of Outcome	Diagnostic Tool	Predictive Value	
						Sens % (95% CI)	Spec % (95% CI)
Caesar et al., 2020 [171]	Australia	Ten studies ≤ 32 weeks GA ± ≤ 1500 g infants (*n*) 992	Sever motor delay (not CP)	2 years	GMA Fidgety stage (AF, F-)	70	85
					HINE at 3 and 6 months	93 (68–100)	100 (96–100)
			Cognitive delay BSID III ≤ 2SD		GMA Fidgety stage (AF, F-)	70	85
					HINE at 3 and 6 months	Not estimable	Not estimable
Novak et al., 2017 [170]	International	Eight studies All GA	CP	<2 years	TEA MRI (preterm infants)	86–89	-
					GMA (Prechtl)	98	-
					HINE	90	-
Bosanquet et al., 2013 [169]	Australia	19 studies 23–41 weeks	CP after 2 years of age	Preschool children (<5 year)	TEA MRI (preterm infants)	86–100	89–97
					Cranial US	74 (63–83)	92 (81–96)
					GMA	98 (74–100)	91 (83–93)
					Neurological examination	88 (55–97)	87 (57–97)

Note. CI: Confidence interval, CP: cerebral palsy, GA: gestational age, GMA: General Movements Assessment, HINE: Hammersmith Infant Neurological Examination, MRI: magnetic resonance imaging, RCT: randomized control trial, SD: standard deviation, Sens: sensitivity, Spec: specificity, TEA: term equivalent age, US: ultrasound, weeks: weeks.

## Data Availability

Not applicable.
